# Chimpanzees use observed temporal directionality to learn novel causal relations

**DOI:** 10.1007/s10329-019-00754-9

**Published:** 2019-09-23

**Authors:** Claudio Tennie, Christoph J. Völter, Victoria Vonau, Daniel Hanus, Josep Call, Michael Tomasello

**Affiliations:** 1 Department for Early Prehistory and Quaternary Ecology, University of Tübingen, Tübingen, 72070 Germany; 2 Messerli Research Institute, University of Veterinary Medicine Vienna, Medical University of Vienna, University of Vienna, Vienna, Austria; 3 School of Psychology and Neuroscience, University of St Andrews, St Andrews, KY16 9JP UK; 4 Department of Developmental and Comparative Psychology, Max Planck Institute for Evolutionary Anthropology, Leipzig, 04103 Germany; 5 Department of Psychology and Neuroscience, Duke University, Durham, NC 27708-0086 USA

**Keywords:** Causal cognition, Social learning, Chimpanzees, Action representation, Simultaneous conditioning, Primate cognition

## Abstract

We investigated whether chimpanzees use the temporal sequence of external events to determine causation. Seventeen chimpanzees (*Pan troglodytes*) witnessed a human experimenter press a button in two different conditions. When she pressed the “causal button” the delivery of juice and a sound immediately followed (cause-then-effect). In contrast, she pressed the “non-causal button” only after the delivery of juice and sound (effect-then-cause). When given the opportunity to produce the desired juice delivery themselves, the chimpanzees preferentially pressed the causal button, i.e., the one that preceded the effect. Importantly, they did so in their first test trial and even though both buttons were equally associated with juice delivery. This outcome suggests that chimpanzees, like human children, do not rely solely on their own actions to make use of novel causal relations, but they can learn causal sequences based on observation alone. We discuss these findings in relation to the literature on causal inferences as well as associative learning.

**Electronic supplementary material:**

The online version of this article (10.1007/s10329-019-00754-9) contains supplementary material, which is available to authorized users.

## Introduction

Learning causal sequences enables organisms to flexibly adapt to their environment and, potentially, to control it (Waldmann and Hagmayer 2005). Experiencing the effects of their own actions is one of the main ways that organisms have at their disposal for learning causal sequences (Leising et al. 2008). Despite its theoretical and practical importance, to what extent nonhuman animals are also capable of learning causal sequences by mere observation (and after minimal exposure) is largely unknown (e.g., Blaisdell et al. 2006; Bonawitz et al. 2010; Taylor et al. 2014; Tomasello and Call 1997; Premack 2007; Völter et al. 2016). Learning causal structures based on correlational evidence is difficult because multiple causal structures can lead to the same pattern of correlations (the “causal inverse problem”; Gopnik et al. 2004). Observing interventions of other agents can be an effective way to learn causal sequences by observation alone (Gopnik and Schulz 2010; Woodward 2012). Particularly, events that reliably follow actions can usually be interpreted as the effect of this action (Meltzoff et al. 2012). Temporal directionality is one of the defining features of causal relations as opposed to correlations (Goldvarg and Johnson-Laird 2001). Keeping track of the temporal order of events when watching the actions of other agents can therefore help to reduce the causal ambiguity.

In nonhuman primates, research on social learning has mostly been concerned with the question to what extent, particularly nonhuman great apes (henceforth: apes), resemble human children with respect to their imitative (especially: action copying) abilities (e.g., Nagell et al. 1993). Whereas apes have sometimes been found to spontaneously copy familiar actions (Fuhrmann et al. 2014; Tennie et al. 2012) they do not appear to copy novel actions (Clay and Tennie 2018; Tennie et al. 2012). It has been suggested that unenculturated, untrained apes instead rely more (or even exclusively) on other social learning mechanisms such as emulation and local enhancement (e.g., Nagell et al. 1993; Tennie et al. 2010). In two-location tasks, in which participants are presented with demonstrations involving manipulations of two different locations on/in a puzzle box, apes (unlike human children) have been found to be selective in their own approach following observational learning opportunities: namely when the apes could see that one of the target locations was visibly not physically connected with the location of the reward, they subsequently ignored that location in their approach (Horner and Whiten 2005; Nielsen and Susianto 2010). In the latter situation, apes tended to manipulate predominantly the target location that was visibly physically connected to the location of the food reward. This raises the question of how apes differentiate between causally relevant and irrelevant location demonstrations in such situations. Apart from physical connectedness, spatio-temporal contiguity and temporal directionality can provide relevant cues. In the two-location task, manipulation of the causally relevant location of the puzzle box directly preceded the outcome (i.e., the appearance of the reward). It is thus possible that apes made use of the temporal structure of events, which has been termed observational causal learning in the developmental literature (Meltzoff et al. 2012).

In the current study, we therefore attempted to replicate a key finding from the developmental literature on observational causal learning (Meltzoff et al. 2012, Experiment 3) in chimpanzees (*Pan troglodytes*). Chimpanzees witnessed a human experimenter press two buttons; pressing one button was immediately followed by the delivery of juice and a sound (cause-then-effect) while pressing the other one was preceded by delivery of juice and a sound (effect-then-cause). If chimpanzees, like 24-month-old human children, can learn directed causal relations from temporal cues alone, we hypothesized, they would prefer the button whose activation preceded the effect (i.e., the cause-then-effect demonstration).

## Materials and methods

### Subjects

We tested 22 chimpanzees housed in two groups at Wolfgang Köhler Primate Research Center, Leipzig Zoo, Germany. Subjects voluntarily participated in the study and were neither food- nor water-deprived. We excluded five subjects for not reaching the training criterion, as described below.

### Apparatus and stimuli

We tested subjects individually in two testing rooms (group A: 25 m^2^; group B: 19 m^2^). Four subjects could not be separated from their respective groups. Consequently, they were tested with conspecifics staying in an adjacent room. However, we occluded the conspecifics’ view on the apparatus and the subject to prevent any observations of the study procedure.

**Fig. 1:**
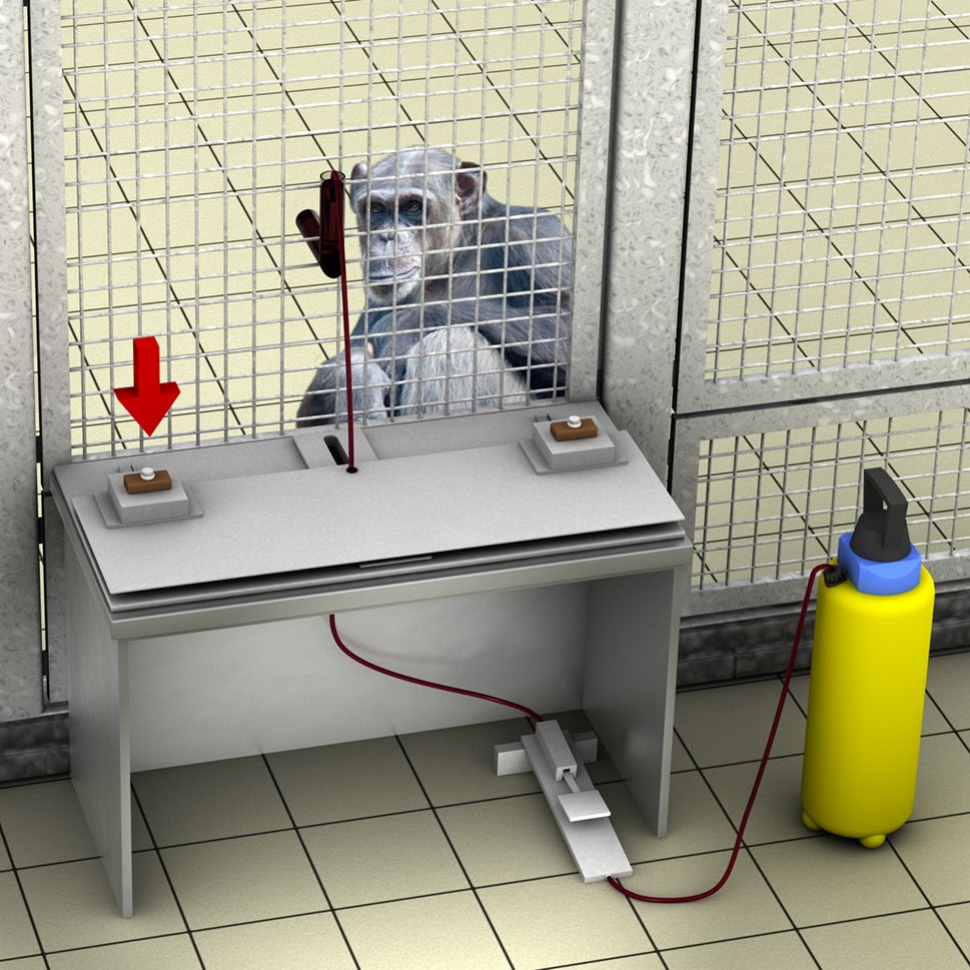
Illustration of the experimental setup. The experimenter’s button press is indicated by the* downwards arrow* (here the left button). For the causal sequence, the onset of the juice flow coincided with the button press—thus appearing to cause the juice flow—while for the non-causal sequence the button press was delayed: here the juice already flowed before the button was pressed. In both cases, the experimenter pressed the button for the same amount of time—thus the juice flow was equally associated with button pressing irrespective of the condition

We videotaped each session. The camera was positioned behind the first experimenter (E1), focusing on the subject as well as on the parts of the apparatus visible to the subject (i.e., the two push-down buttons). The apparatus was attached outside the subject’s testing room and consisted of a sliding table (3 × 80 × 38 cm) with two buttons (mounted on the right and the left side of the sliding platform), a juice dispenser (45 × 17 × 17 cm) below, and three occluders (see Fig. 1). The juice dispenser was an electric liquid garden pump that was altered to pump juice with a constant pressure into the subject’s enclosure once E1 pressed a hidden foot pedal. The delivery of juice was electronically linked to a sound emitter with a tonal sound (approximate frequency of 1 Hz and loudness of 70 dB at 1 m distance) that was audible as long as the hidden foot pedal was pressed (compare Meltzoff et al. 2012). We fixed the sliding table, at a height of 50 cm, to a metal mesh grid (55 × 72 × 2 cm) of the subject’s enclosure.

Four visually distinct pairs of identical buttons served as stimuli. For the training phase, we used a sliding platform with only one centrally placed button on it. This training button was differently shaped and sized than those used for the subsequent observation and test phase. All buttons were fixed on square pieces of plastic that fit flush into square plastic frames (10 × 10 cm) on the sliding platform. We adjusted each button to the same height and distance to the edge of the plastic frame (ensuring that both buttons were located at equal distance from the subject). We painted each button with a thin blue or white ring at its bottom that became invisible when the bottom top was pressed down. This was to facilitate the visual coding of button-pressing.

As a matter of fact, pressing the buttons did not actually lead to any effect—all buttons were sham-buttons—and so E1 produced all effects with the help of pressing the hidden foot pedal. At the beginning of each trial, E1 placed two buttons (one at the upper left and one at the right corner of the sliding platform). E1 exchanged these buttons after each trial for different ones (original placement as well as button exchanges happened behind two occluders). Below the sliding table another occluder (50 × 70 × 35 cm) constantly hid the foot pedal of the juice dispenser as well as E1’s leg movements that activated the pedal. In the middle of the mesh grid, we fixed a Plexiglas tube (inner diameter 3 cm), which went into the subject’s cage to facilitate the drinking for the subject and to make sure that the subject was orientated towards the demonstrations as well as having full view of them. This tube also was connected to and supported (and protected) the hose that went through a hole in the middle of the sliding table to connect to the juice dispenser setup. E1 adjusted a hose coupling prior to trials so that the amount of delivered juice stayed constant over all trials (i.e., 50 ml per single delivery).

### Procedure

There were three phases: a training phase, an observation phase, and a test phase. Following the training phase, we tested subjects in two sessions, with two trials each. The buttons that appeared causal (see below) stayed on the same side within sessions, but were switched across sessions. In every test trial, we presented subjects with a novel pair of buttons.

#### Training phase

Each subject completed three training sessions to ensure that general button-pressing behavior would occur in the test phase. We trained subjects to press a single (central) training button with enough force so that it made an audible click sound. We rewarded them by four continuous seconds of juice (accompanied by the sound) through the juice delivery setup every time they pressed the button. Subjects passed training phase A as soon as they reliably pressed the training button ten times in a row in a session that lasted for a maximum of 20 min). This procedure was repeated three times at most. Subjects reached the training criterion if they further managed to press the training button ten times in a row within 60 s during two subsequent sessions (training phase B). Once a subject passed training phases A and B, we presented it with the observation and test phase. Seventeen subjects passed and five subjects failed to reach this criterion. We excluded the latter individuals from further testing. Before each of the two test sessions (i.e., before trial 1 and 3), we performed refresher trials, in which subjects had to press the centralized single training button two times within 60 s. All subjects passed these refresher trials.

#### Observation phase

The observation phase largely followed the procedure administered by Meltzoff et al. (2012, Experiment 3). The causal sequence (“causal button”; henceforth “C”) started with a prolonged manual button press whose initiation coincided with juice delivery and sound. E1 released the button after 2 s, while the sound emitter and juice dispenser were activated for another 2 s (i.e., 4 s in total). For the other “non-causal button” (henceforth “N”), we reversed the sequence, i.e., the activation of the juice dispenser and sound emitter preceded the button press (but juice/sound lasted again 4 s). During N demonstrations E1 pressed the button coinciding with the start of the third second of juice flow and sound for a duration of 2 s. At the beginning of each trial, subjects received ten demonstrations in total (five per C button and five per N button), during which we kept both buttons out of the subject’s reach.

The demonstration order was taken from Meltzoff et al. (2012) and followed either a CNNCNNCNCC (henceforth “Version 1”) or a NCCNCCNCNN (henceforth “Version 2”) design—counterbalanced across subjects. Half of the subjects started with Version 1 and the other half started with Version 2. Within these two groups, half of the subjects started with a demonstration of the left button first and half of them started with the right button first. We matched the resulting four groups as much as possible for age, rank, and sex. The button types for each trial were identical across the subjects.

E1 always pressed the buttons with the index finger and timed button pressing with the pressing of the foot pedal (either at the same time as juice/sound, in C demonstrations; or with a 2-s delay after juice/sound onset, in N demonstrations) with the help of a metronome. The metronome was audible only for E1 via headphones (one beep per second), which allowed E1 to time the 2- and 4-s intervals. To ensure a reliable timing, E1 passed a practice phase before the start of data collection and fulfilled a 90% correctness criterion after 25 practice trials (a deviance of more than half a second was counted as incorrect).

Mistakes made by E1 in the observation phase led to an immediate repetition of the affected demonstration (i.e., the single affected button demonstration). Overall, these mistakes happened six times: one mistake was made in demonstration order (in a first trial of the first session); five mistakes concerned experimental setup problems (i.e., short-term malfunctioning of the juice delivery system; this happened four times in the first trial of the first session and once in the first trial of the second session). During demonstrations, another experimenter (E2) live-coded whether the subject was attentive during each demonstration (eyes open and gazing towards demonstrations). If E2 deemed the subject to have been inattentive, only the single affected button demonstration was repeated. This happened five times in total (once in trial 1 and once in trial 2 of the first session and once in trial 1 and twice in trial 2 of the second session). Once the subject had attended to the required five demonstrations per button (causal and non-causal)—ten in total—the test phase commenced.

#### Test phase

The test phase immediately followed the observation phase. For the test phase, E1 moved the sliding platform with the two buttons (the same ones used during the observation phase) towards the mesh grid, enabling the subject to press one of the buttons. A button press was coded once the subject pressed with enough force so that the trigger markings became invisible and the ‘click’ sound was audible. Subjects were rewarded non-differentially: any button press immediately released juice (and produced the sound) for four continuous seconds (controlled always by E1 pressing the foot pedal coinciding with the button press as defined above). If the subject failed to press any of the buttons within 60 s, the demonstration phase was repeated on a different day and this test trial was then also repeated [this happened three times in total (all in session 1: twice in the first trial and once in the second trial)]. After the subject had pressed a button, the sliding table was pulled back to prevent the subject from ‘double’-choice (i.e., from pressing the other button). Double-choices could not be fully prevented in this way, but they only happened five times across all subjects and sessions (twice in session 1, both in the first trials of the session), and three times in session 2 (two of which happened in the first trials of the session, and one in the second trial). Double-choices were not rewarded.

### Scoring

The data of 17 subjects (who passed the training phase) were analyzed. All statistical tests were two-tailed. E1 coded from the video, which button was pressed (button choice), defined as producing a click sound and making the painted line below the button top invisible.

The mean performance in the 11 trials that included a repeated demonstration (due to experimenter mistakes or subjects’ inattentiveness) during the observation phase (64% correct) was similar to the performance in the remaining trials (58% correct). Crucially, subjects’ above-chance performance in the first session was not driven by these trials (trials with a repeated demonstration: 67% correct; remaining trials: 82% correct).

A second coder blind to the purpose of this study coded button choice for 50% of the trials, randomly chosen. Inter-rater agreement was excellent (Cohen’s kappa = 0.88, *N* = 34).

### Analysis

We fitted a generalized linear mixed model (GLMM; Baayen 2008) with binomial error structure and logit link function (McCullagh and Nelder 1989) to analyze the influence of the predictor variables session, trial number (within session), and the side of the causal button on chimpanzees’ choice performance (causal button presses coded as “1”, non-causal button presses coded as “0”). We included subject ID as random effect and all possible random slope components (Barr et al. 2013). Following a significant effect of session, we analyzed the two sessions separately. Specifically, we used the intercept of these models to evaluate whether the performance in each session deviated significantly from chance level of 0.5 while accounting for trial number and the side of the causal button.

As an overall test of the effect of the predictor variables, we compared the full model with a null model lacking the test predictors but comprising the same random effect structure as the full model (Forstmeier and Schielzeth 2011) using a likelihood ratio test (Dobson 2002). *P* values for the individual effects were based on likelihood ratio tests comparing the full with respective reduced models (Barr et al. 2013) using R function drop1 with argument ‘test’ set to “Chisq”. The model was implemented in R (version 3.5.2; R Development Core Team 2018) using the function glmer of the R package lme4 (Bates et al. 2016). Confidence intervals were derived using the function bootMer of the R package lme4, using 1000 parametric bootstraps and bootstrapping over the random effects.

Prior to fitting the models, all predictor variables were z-transformed (to a mean of zero and a standard deviation of one). We determined variance inflation factors (Field 2005) for standard linear models excluding the random effects using the R package car (Fox and Weisberg 2011). Collinearity was no issue (maximum variance inflation factor: 1.00).

## Results

The full model fitted the data significantly better compared to a null model that lacked the predictor variables but included the same random effects structure (*χ*^2^ = 8.58,* df* = 3, *p* = 0.035). Chimpanzees performed significantly better in the first session compared to the second session (for the model results, see Table 1). There were no significant effects of trial number (within session) or the side of the causal button.

**Table 1:** Results of the GLMM with causal button choices as response variable

	Estimate	SE	*χ*^2^	*p*	95% CI	
Intercept	0.659	0.393			− 1.061	11.36
Session	− 1.544	0.661	7.457	0.006	− 21.145	− 0.687
Trial number	0.206	0.458	0.243	0.622	− 3.519	5.674
Side of causal button	0.180	0.608	0.085	0.770	− 4.362	9.393

The predictor variables were z-transformed to a mean of zero and a standard deviation of one

Following the significant effect of session, we analyzed the two sessions separately. We found that chimpanzees performed significantly better than expected by chance in session 1 (intercept: estimate ± SE: 6.71 ± 2.42, *z* = 2.78, *p* = 0.006, 95% CI [2.39, 12.64]) but not in session 2 (intercept: − 0.53 ± 0.42, *z* = − 1.26, *p* = 0.207, 95% CI [− 12.59, 0.67]).

**Fig. 2:**
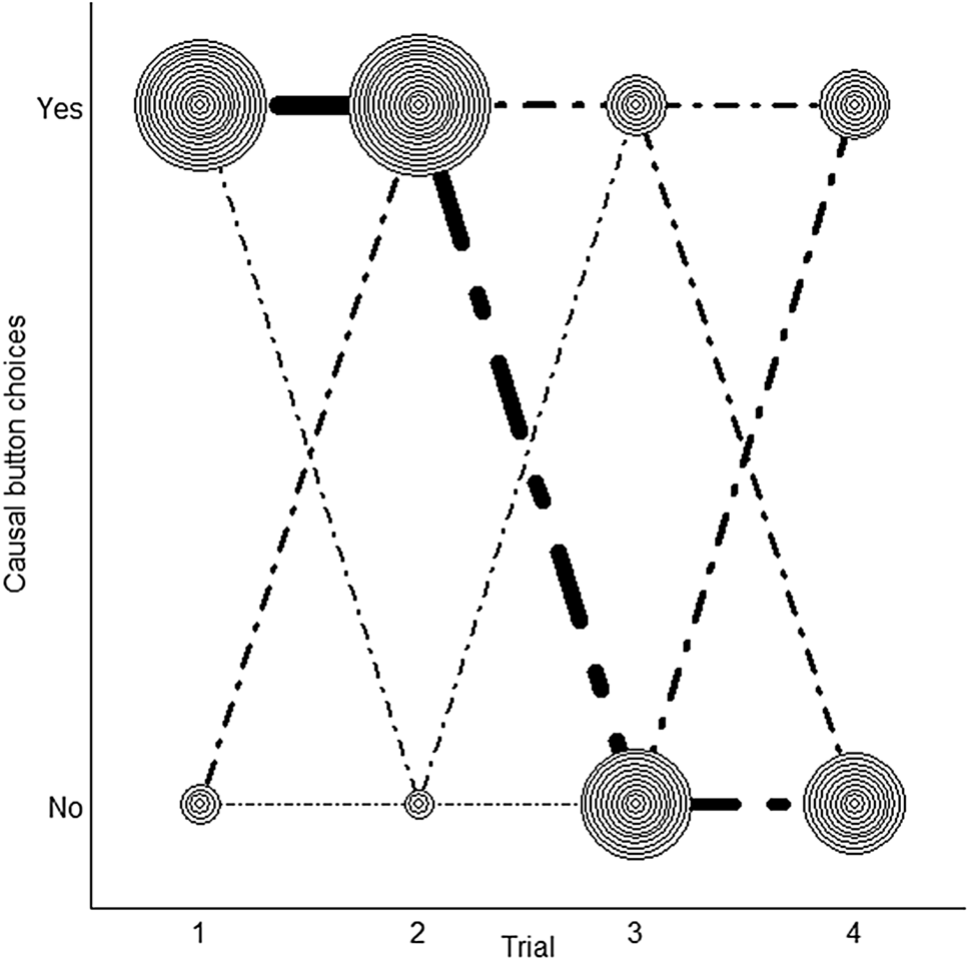
Causal button choices as a function of trial number. Each* circle* corresponds to one individual. The width of the lines that connect the circles is proportional to the number of represented individuals. Individuals who consistently chose the causal button in session 1 (trials 1 and 2) tended to choose the same side in session 2 (trials 3 and 4, resulting in non-causal button choices due to the administered counterbalancing scheme)

We found a similar pattern when we analyzed the data on a trial-by-trial basis (see Fig. 2). In both trials of the first testing session, the chimpanzees chose the causal button, as predicted, significantly more often than the non-causal one (trial 1: 13 of 17 chimpanzees chose the causal button; exact binomial test, *p* = 0.049; trial 2: 14 of the 17 chimpanzees chose the causal button; exact binomial test, *p* = 0.013). During the second session and contrary to our predictions, subjects’ preference for the causal button disappeared (trial 3: six out of 17 chimpanzees choose the causal button, exact binomial test: *p* = 0.33; trial 4: seven of 17 chimpanzees choose the causal button, exact binomial test: *p* = 0.63).

Twelve of the 17 chimpanzees chose the same side twice in session 1. Ten of these 12 chimpanzees chose the same side again in their first trial in session 2 (exact binomial test: *p* = 0.039; see Fig. 2), suggesting that the majority of the chimpanzees acquired a side bias in session 1.

## Discussion

After the demonstrations, when given the opportunity to activate the juice dispenser themselves, chimpanzees in session 1 preferentially pressed the causal button, i.e., the one that preceded the effect. Importantly, they did so immediately in their first trial even though both buttons were equally associated with juice delivery, i.e., both button presses completely overlapped (by 2 s) with the juice flow. These results suggest that chimpanzees use temporal information to distinguish observed interventions that predict an outcome from actions that follow an outcome. This finding confirms that chimpanzees are sensitive to the temporal order of causal sequences (Hanus and Call 2008; Völter and Call 2014).

However, while the temporal order of events initially influenced chimpanzees’ choices, our findings also suggest that this learning bias might not prevail over the reinforced location. Chimpanzees who consistently chose one side in session 1 preferred to pick the same side in the first trial of session 2, even though the causal and non-causal button positions were now reversed. The decrease in performance between session 1 and 2 might therefore be an artefact of the applied counterbalancing scheme. This outcome suggests that when presented with a novel situation, chimpanzees pay attention to the temporal order of events. However, when they encounter the same situation again, they might remember the side of their previous response that led to the reward without paying attention to the temporal information provided in the current demonstration. The non-differential reinforcement might have contributed to the acquisition of this side bias.

Our interpretation of the results is in line with two studies that investigated causal learning in great apes. In these studies, the chimpanzees performed particularly well (and significantly above chance) in the first trial of the experimental condition (Völter and Call 2014; Völter et al. 2016). In contrast to the present study, however, in those studies the side of the correct option was not blocked within each session, which might explain why they did not acquire a side bias in those studies. Additionally, a number of studies on problem-solving abilities suggest that chimpanzees tend to stick to the first acquired problem-solving strategy (Gruber 2016; Hrubesch et al. 2009; Marshall-Pescini and Whiten 2008), as long as it leads to the acquisition of the food reward (Manrique et al. 2013). Together, these studies support the idea that chimpanzees pay attention to the temporal order of events when they encounter novel situations, which, in turn, biases their choice performance. At the same time, the resulting positive reinforcement can lead to the acquisition of a side bias rather than increased attention to the temporal structure of events.

One might argue that we trained chimpanzees in the training phase to choose a causal button because they experienced that pressing a button (presented centrally in the training phase) preceded delivery of juice. This account is not at odds with our interpretation of the data: after limited experience with a novel means–ends relationship in this training phase, chimpanzees made differential use of the temporal sequence in an observational learning context with two novel stimuli (i.e., novel buttons) and they distinguished button presses contingent with the onset of the reward from button presses that occurred after the onset of the reward.

The temporal sequence of events also seems to play an important role in associative learning. If a conditioned stimulus (CS), such as a light, is forward paired with an unconditioned stimulus (US), such as a footshock, animals tend to show stronger conditioned responses than when the US precedes the CS or when CS and US are presented simultaneously. Traditionally, this has been interpreted as evidence that learning is faster in forward conditioning than in backward or simultaneous conditioning (Pavlov 1927). However, more recent evidence suggests that associations are formed irrespective of the timing of the CS presentation (e.g., Barnet et al. 1991; Matzel et al. 1988). Instead, the difference between forward conditioning and backward/simultaneous conditioning seems to be whether animals anticipate the US based on the CS presentation, which appears to be the case particularly in forward conditioning. Savastano and Miller (1998) therefore concluded that animals encode the temporal relationship between events which affects the expression of the anticipatory response. The integration of temporal information in rats seems not to be restricted to fear conditioning but has also been reported in a appetitive conditioning context (Leising et al. 2007). The results of the present study supports these conclusions and extend it to nonhuman primates. Nevertheless, our findings differ in multiple ways from the previous work with rats: first, apes made a choice in the test phase between two CS, which had been previously paired in different temporal arrangements with the US. In contrast, in rats the strength of the conditioned response was compared between subjects (following different types of CS—US pairings; Barnet et al. 1991; Matzel et al. 1988). Second, we found differences between the simultaneous presentation of the CS and US and the US ⟶ CS backward pairing. In contrast, no marked difference between backward and simultaneous conditioning has been reported in rats (see Matzel et al. 1988).

One might argue that the difference between conditions was due to chimpanzees being distracted by the (juice) reward during the non-causal demonstration. In particular, during the non-causal button demonstration, chimpanzees were drinking juice when the experimenter pressed the non-causal button, which was not the case for the causal button. This in turn might have led to a weaker association between the non-causal button and the juice delivery compared with the causal button. However, our experimental design ensured that there was no obvious difference in overt attention between the two conditions. A second experimenter coded whether chimpanzees were attentive in both conditions and trials were repeated when chimpanzees were not watching the demonstration.

In sum, our results suggest that chimpanzees, like human children (see Meltzoff et al. 2012), can efficiently learn novel causal relations based on the temporal directionality of external events. Future studies are needed to determine the stability and flexibility of chimpanzees’ causal learning and which factors might enhance it, or inhibit it. With regard to stability, our study tentatively suggests that chimpanzees’ competence in this regard might be rather fragile and/or easily overridden. With regard to flexibility, one intriguing question for future research is to what extent chimpanzees can also use observation to infer more complex causal structures, including the use of observation to disambiguate more complex causal structures, similar to the work that has been done with children (Gopnik et al. 2001; Sobel et al. 2004; Völter et al. 2016).

## Electronic supplementary material

Below is the link to the electronic supplementary material.

Supplementary material 1 (XLS 51 kb)
